# Transcriptional Control of Th9 Cells: Role of Foxo1 in Interleukin-9 Induction

**DOI:** 10.3389/fimmu.2018.00995

**Published:** 2018-05-09

**Authors:** Sakshi Malik, Amit Awasthi

**Affiliations:** Immuno-Biology Laboratory, Center for Human Microbial Ecology, Translational Health Science and Technology Institute, Faridabad, India

**Keywords:** T helper cells, T helper 17 cells, inflammation, Foxo1, interleukin-9

## Abstract

Interleukin (IL) 9-producing helper T (Th) 9 cells play a major role in contributing immunity against extracellular pathogens. In addition, the role of Th9 cells was demonstrated in the pathogenesis of allergic, skin, and intestinal inflammation. The functions of Th9 cells were further extended in antitumor immune response, as Th9 cells were suggested to be potent antitumor Th cells. Given the pleotropic functions of IL-9 in various pathophysiological conditions, it is essential to understand the differentiation and stability of Th9 cells and other IL-9-producing T cells. In addition to Th9 cells, Th2 and Th17 cells as well as induced Foxp3^+^ regulatory T cells (iTregs) cells also produce IL-9, but how IL-9 production is regulated in these cell types is not yet clearly defined. Although Th2, Th9 and Th17 cells as well as iTregs develop in the presence of distinct differentiating factors, yet they all express IL-9 together with their own lineage specific cytokines. Here, in this review, we summarize the current understanding of signaling pathways that lead to the promotion of differentiation of Th9 cells and IL-9 induction in Th2 and Th17 cells, as well as in iTregs. We further discuss the transcriptional regulation of Th9 cells in context of Foxo1, as an essential transcription factor required for the development and functions of Th9 and other IL-9-producing T cells.

## Introduction

Almost more than two decades ago, interleukin (IL)-9 was described as T cell growth factor, which was later categorized as one of the Th2 cytokine (1, 2). After the association of IL-9 with Th2 cells was established, much of its functions was tested in Th2-biased mouse models of allergic inflammation and *Leishmania* infection, which further reinforced its classification as Th2 cytokine (3, 4). The functions of IL-9 was not greatly discussed separately, as it was thought to be enhanced during disease pathology induced by Th2 cells. Nonetheless, the genetic association studies identified the association of IL-9 and IL-9R with human asthma, which was further validated in mouse model of allergic inflammation in asthma (5, 6). Pulmonary overexpression of IL-9 was seen to be associated with inflammatory infiltration of eosinophils and lymphocytes (7). One of the striking findings in this model was greatly enhanced mast cell infiltration within the airway epithelium. This was in agreement with other findings which identified that lung-expression of IL-9 increased IgE-mediated disease pathology and mucus production in mouse model of asthma. These observations were further validated in transgenic mice in which lung-specific inducible IL-9 production was controlled by doxycycline (8). Consistent with constitutive expression of IL-9, doxycycline inducible IL-9 production in the lung promotes lymphocytic and eosinophilic infiltration with mucus production and mast cell hyperplasia, which leads to lung immune-pathology (8). In addition, IL-9 overexpression further enhanced the production of Th2 cytokines such as IL-4, IL-5, and IL-13. Strikingly, neutralization of IL-13 leads to inhibition of both lung inflammation and mucus production resulting in suppression of lung immune-pathology in allergic inflammation. In order to further refine the functions of IL-9 in comparison to other Th2 cytokines, IL-9-deficient mice were generated. IL-9-deficient mice manifest highly defined phenotype of Th2 responses such as mast cells proliferation and mucus production without affecting worm expulsion (6).

The clarity in IL-9 functions in immune responses came with identification and discovery of IL-9-producing Th9 cells (9, 10). It was identified that the activation of naïve T cells in the presence of TGF-β1 together with IL-4 induced the generation of IL-9-producing helper T (Th) cells, and therefore these cells were referred to as Th9 cells (9, 10). While TGF-β1 alone induces Foxp3 expression and generated immunosuppressive Foxp3^+^ induced Tregs (iTregs), addition of IL-4 suppressed TGF-β1 induced Foxp3 expression (9). On the other hand, TGF-β1 suppressed IL-4 functions, which is otherwise known to induce the differentiation of Th2 cells. While TGF-β1 and IL-4 suppressed each other’s respective functions such as Foxp3 induction and Th2 differentiation, but two cytokines together induced a new pathway of Th9 cell differentiation. GATA3 is a common transcription factor of two IL-9 producing sister populations, i.e., Th2 and Th9 cells and one of the major function of GATA-3 in Th9 cells is to counteract the TGF-β1-induced Foxp3 expression, which in turn limit the ability of GATA-3 to induce *Il4* expression (9). Later on, it was identified that other cytokines such as IL-2, IL-1, IL-25, IL-33, IL-7, and TSLP further enhanced the differentiation of Th9 cells induced by TGF-β1 and IL-4 (11–16).

## Differentiation and Transcriptional Regulation of Th9 Cells

The regulatory network of transcription factors in Th9 cells seems to be quite complex, as Th9 cells express number of transcription factors. Nonetheless, classification of a unifying master transcription factor is still ambiguous, as most of the transcription factors expressed in Th9 cells is also co-expressed by other T helper lineages. In order to simplify the complex network of Th9 cell transcription factors, the different transcription factor involved in Th9 cells development can be distributed into different groups depending upon their priming signals. For example, downstream of TGF-β1, Smad-dependent pathway majorly regulates RBP/Notch signaling while TAK1-mediated Smad-independent pathways control the induction of Id3 and HIFα in Th9 differentiation (17–19). PU.1, which is one of the major transcription factor, is regulated by TGF-β1, and is not dependent on Smad2/3 (20). Although IL-4–STAT6 signaling seems to regulate BATF/IRF-4 and ETV5 in Th9 cells, TGF-β1 also enhances binding of IRF-4 to *Il9* locus (21–23). In addition to IRF-4, other interferon regulatory factors such as IRF-1 and IRF-8 are also involved in IL-9 regulation in Th9 cells (24–26). While IL-1β induces IRF-1, TGF-β/Smad3 pathway induces IRF-8 in Th9 cells (24, 26). T cell receptor (TCR)-dependent signals regulate the function of NFAT, TNF superfamily, NF-κB, and Foxo family members in various T cell subsets (27–30, 62). It may be possible that these factors work in a concerted manner to drive optimal differentiation of Th9 cells, however, complex regulatory network of Th9 cells is not yet defined. Nonetheless, a recent report has identified as to how IRF-8 cooperatively interacts with IRF-4/BATF/PU.1 to promote Th9 development while simultaneously repressing *Il4* transcription, suggesting the involvement of large molecular transcriptional complexes in Th9 differentiation akin to Tregs (26, 31).

PU.1, an ETS family transcription factor is one of the first factors that were seen to be specifically associated with IL-9 induction in Th9 cells (32, 33). PU.1 imprints heterogeneity in Th2 cells in respect of IL-9 induction, as overexpression of PU.1 increases IL-9 production with concomitantly decreases type-2 cytokines production. Molecularly, PU.1 restricts the ability of GATA3 and IRF-4 to induce Th2 cytokines signature, and thereby promoting the differentiation of low level of IL-4 production in Th2 cells. Furthermore, mice with PU.1 deficiency in T cells develop attenuated allergic inflammation in lungs in response to OVA, which is found to be associated with reduced amounts of IL-9 production and Th2 cytokines. In addition, PU.1-dependent IL-9 induction was linked to the pathology of intestinal inflammation, as IL-9 deficient as well as *Spi* conditional deficiency in T cells were seen to have reduced clinical and histological signs in oxazolone-induced colitis model (33).

In addition to PU.1, another ETS family member, ETV5, exerts dominant effect on IL-9 induction in Th9 cells, as ETV5-deficient T cells have shown reduced *Il9* expression. Consistently, ectopic expression of ETV5 enhanced induction of IL-9 in Th9 cells (23). Interestingly, IL-9 production from Th9 cells was found to be further suppressed upon a combined deficiency of both PU.1 and ETV5 as compared to either PU.1 or ETV5 single deficiency, suggesting that PU.1 and ETV5 work in concerted manner to induce optimal IL-9 induction and Th9 cell differentiation. Although ETV5 and PU.1 belong to the same family, their induction and functions differ in Th9 cells. While PU.1 was shown to be induced by TGF-β1 signaling, ETV5 was found to be induced and essential for IL-4–STAT6 axis in Th9 differentiation. Mechanistically, ETV5 physically binds to *Il9* locus at sites that are distinct from PU.1 DNA-binding motif, and transactivate IL-9 induction in Th9 cells. The functions of other ETS family member such as Elk3 and Etv6 were also tested in Th9 cells differentiation, but found not be essential for IL-9 induction in Th9 cells. This implies that among other ETS family member, ETV5 and PU.1 play selective and specific role of in Th9 differentiation.

Although the role of IRF-4 has been identified in the development and functions of Th2 and Th17 cells, it has been shown that IRF-4 is essential for differentiation of Th9 cells (22). In fact, both BATF and IRF-4 have been shown to work cooperatively in Th9 cells development, as deficiency of BATF have shown to reduce the binding of IRF-4 to *Il9* promoter or *vice-versa* (21). Consistently, ectopic expression of BATF failed to rescue IL-9 production in the absence of IRF-4 in Th9 cells (21). Similar to PU.1-deficient mice, IRF-4-deficient mice display attenuated signs of development of IL-9-dependent OVA-induced allergic inflammation in lungs in mouse model of asthma (21). Molecularly, chromatin immunoprecipitation (ChIP) sequencing analysis combined with proximity ligation assays have identified that, in addition to BATF-IRF-4 complex, IRF-8 interact and form large transcriptional complexes with IRF-4/BATF/PU.1 to induce the development of Th9 cells (26). Interestingly, it has been proposed that IRF-8 executes dual functions in Th9 cells differentiation, while on the one hand, it partners with other transcription factors to form large transcriptional complex to optimally induce IL-9, on the other hand, IRF8/Etv6 heterodimer represses *Il4* transcription. Taken together, these observations clearly indicate the dual functions of IRF-8 in promoting Th9-exclusive gene signature. In addition to IRF-4 and IRF-8, the involvement of IRF-1 and its functions in Th9 differentiation remains unclear, as two independent studies have reported contrary functions of IRF1 in Th9 development (24, 25). While Végran et al. have shown that IRF1-deficient CD4^+^ T cells have profound defect in Th9 differentiation, on contrary to this Campos Carrascosa et al. have identified that IFN-γ-induced IRF1 promotes transcriptional shift of Th9 cells to Th1 cells, as IRF1 outcompetes IRF4 binding at *Il9* promoter. The discrepancy in these two studies could be accounted to the cytokines used for inducing Th9 differentiation. Végran et al. used IL-1β together with TGF-β plus IL-4 to induce Th9 differentiation, which leads to the induction of IRF-1. Campos Carrascosa et al demonstrated that IFN-γ/IRF1 axis negatively regulates the differentiation of Th9 cells induced by TGF-β plus IL-4 (24, 25).

In addition to the cytokines induced transcription factors, TCR-stimulated activation of NFAT and NF-κB contributes rapid induction of IL-9 in Th9 cells. Both NFAT1 and NF-κB work together synergistically in Th9 differentiation. NFAT1 facilitates the binding of NF-κB p65 to *Il9* promoter by actively remodeling chromatin, as T cells-deficiency of NFATc1/NFATC2 produces attenuated IL-9 in mouse model of allergic inflammation (27). Two major components of NF-κB pathway, i.e., RelB-p52 and p50 are induced upon OX-40 and GITR ligation, respectively, in Th9 cells (28–30). In addition to TCR-mediated activation of transcription factors, ligation of secondary co-stimulatory checkpoint molecules on Th9 cell enhance the development of Th9 cells by further promoting the activation of transcriptional network that support Th9 differentiation. While OX-40, a member of TNFR superfamily of receptor, induced IL-9 is dependent on STAT6, GITR induces the activation of STAT6, BATF, PU.1, and IRF-4 in Th9 cells (28–30). Interestingly, GITR ligation enhances IL-9 expression in Th9 cells in the absence of IL-4 receptor signaling but not in STAT6 deficient mice, as induction of STAT6 under GITR stimulation is IL-4–IL-4R is independent and is required for Foxp3 repression (29). Surprisingly, other TNFRSF member, TLA1/DR3 requires functional IL-2/STAT5 pathway but is independent of NF-κB or STAT6 in Th9 cells (34).

Engagement of TGF-β with its receptor activates Smad-dependent and -independent pathways that leads to Th9 differentiation. It has been shown that T cells lacking Smad2, Smad3, or Smad4 (Smad2^fl/fl^CD4^cre^, Smad3^−/−^ T cells and Smad4^fl/fl^ CD4^cre^ animals) have reduced IL-9 production in Th9 cells (20). Mechanistically, Smad-deficiency leads to modifications in histone acetylation/deacetylation and methylation at *Il9* promoter or CNS regions, suggesting that Smads might be essential for favorable epigenetic modifications of *Il9* locus in Th9 cells (20). In fact, as compared to single gene deficiency of either Smad2 or Samd3, double deficiency of Smad2/Smad3 leads to profound reduction in IL-9 production in Th9 cells, which found to be associated with reduced histone acetylation marks in *Il9* locus (20). In addition, it has been found that Smad3 bind at a site near to recombination-signal-binding protein for immunoglobulin-κ-J region (RBP-Jκ) and the Notch intracellular domain in IL-9 locus to further positively regulate the differentiation of Th9 cells (17). In addition to Smad dependent pathway, TGF-β1-induced Smad-independent pathways are primarily coordinated *via* TAK1, as inhibition of TAK1 suppresses two major transcriptional repressors, Id3 and SIRT1, in Th9 cells developmental pathway (18, 19). Id3, an E-box transcription factor inhibitor, act as a negative regulator in Th9 differentiation. Molecularly, Id3-deficient T cells have shown an increased binding of E2A and GATA-3 at *Il9* promoter, suggesting that the absence of Id3 promotes accessibility of *Il9* locus to Th9-associated transcription factors leading to Th9 differentiation. Similar to Id3, SIRT1-deficient T cells have increased IL-9 production due to enhanced HIF-1α-dependent glycolysis in Th9 cells (19).

In addition to factors induced by TGF-β1, IL-4, and TCR, IL-2–IL-2 receptor pathway plays a critical role in enhancing IL-9 production and Th9 development (11). Upon binding to its receptor, IL-2 induces STAT5 activation that lead to differentiation of Th9 cells. Moreover, nitric oxide, Itk activation, TSLP, and TL1A enhanced IL-9 induction in Th9 cells is dependent on IL-2 (34–36). Although activated STAT5 binds directly to *Il9* promoter to induce IL-9 gene expression, however, the functions of STAT5 in Th9 cells are suppressed by BCL-6. Mechanistically, BCL6 competes with STAT5 for binding at the *Il9* promoter in Th9 cells, thus suppresses the development of Th9 cells (11). In addition to STAT5, IL-1β-induced STAT1 was also found to amplify IL-21 and IL-9 production *via* STAT-1/IRF-1 module to enhance antitumor functions of Th9 cells (24).

## Physiological Importance of Th9 Cells

Physiologically, Th9 cells were shown to play crucial roles in aggravating inflammation in disease like asthma, EAE, colitis, and skin inflammation (37, 38). IL-9 and IL-9R single nucleotide polymorphisms (SNPs) have been associated with allergen sensitization in allergy. Other Th9-associated genes such as IL-4RA, STAT6, and IL-33 are also found to be associated with allergic inflammation in human diseases (5, 39–41). Consistently, administration of anti-IL-9 neutralizing antibody in murine models of asthma decreased the severity of disease associated with attenuated infiltration of eosinophils and AHR, suggesting a crucial role of IL-9 in progression of allergic inflammation in asthma (15, 21, 22). Nevertheless, humanized anti-IL-9 neutralizing antibody (MEDI-528) clinical trial could not report any improvement in subjects as compared to control treatment (42). This could be attributed to the polygenic nature of asthma and genetic basis of heterogeneity in different individuals. Since asthma is contributed by waves of different cytokines such as IL-4, IL-5, IL-13, and IL-9, hence it is needful to define and identify the predominant cytokine signature and subtype that is inducing the disease.

IL-9 and Th9 cells were found to be associated with skin inflammation. Human IL-9-producing Th9 cells were found to be skin tropic and express cutaneous lymphocyte antigen (CLA), which by virtue makes them skin tropic (38). Further analysis identified that skin tropic CLA^+^ human Th9 cells were found to be independent of TGF-β1 and IL-2, and were accumulated in psoriatic lesions (38). It is, however, intriguing as to what is the functions of Th9 cells in skin under homeostatic conditions. Whether Th9 cells are required for the maintenance of barrier functions in the skin surfaces are not yet elucidated. Based on the observations that IL-9 can acutely stimulate mast cells, the constant presence of Th9 cells under the skin might potentially activate innate immune cells including mast cells upon skin infection to contain pathogens. In fact, IL-9 is shown to induce IL-8 production from keratinocytes, which promote the influx of neutrophils at the site of fungal infections (43, 44). Emerging literature is suggesting that IL-9 potentially contributes to different types of skin disorders such as atopic dermatitis, allergic contact dermatitis, allergen-induced delayed type hypersensitivity, psoriasis, and cutaneous T cell lymphoma (45).

In the gut inflammation in inflammatory bowel diseases (IBD), IL-9-producing CD4^+^ T cells were found to be colitogenic, as gut epithelial cells of ulcerative colitis patients expressed elevated levels of IL-9R. Moreover, lamina propria T cells from IBD patients were found to have an increased frequency of CD4^+^PU1^+^IL-9^+^ and CD4^+^IRF4^+^IL-9^+^ T cells, suggesting a strong association of IL-9 with disease severity in IBD (33, 46). In mouse models of colitis, adoptive transfer of *in vitro* differentiated Th9 cells into Rag-deficient hosts led to the development of severe colitis in an IL-9 dependent manner (33, 47, 48). In chemically induced model of colitis, Th9 cells were found to be one of the major effector T cells population that induced disease pathogenesis, as both PU.1- and IL-9-deficient mice were found to have reduced incidence of colitis and reduced inflammatory score as compared to wild-type mice (33). Consistently, treatment with anti-IL-9 neutralizing antibody was found to effectively control tissue inflammation of intestine in colitis (33). Mechanistically, IL-9 was found to suppress epithelial cell proliferation thereby affecting mucosal wound healing in IBD. In fact, topical administration of IL-9 was found to repress epithelial cells tissue repair mechanism *in vivo* (33). Nonetheless, the protective role of IL-9 was also attributed in DSS-induced colitis, as NKT cells-driven IL-9 is found to protect gut inflammation in DSS-induced colitis (46).

In addition to the intestinal inflammation, Th9 cells were found to be associated with tissue inflammation in EAE, a mouse model of human multiple sclerosis. IL-9^+^ T cells can be isolated from the draining lymph nodes of mice that develop EAE. In fact, similar to Th1 and Th17 cells, adoptive transfer of MOG-specific Th9 cells into Rag-deficient mice induced the development of EAE (49, 50). Nonetheless, CNS lesions induced in Th9 transfer model were quite different in their appearances as compared to Th1 and Th17 cells transfers (51). In addition to inducing tissue inflammation in autoimmune diseases, IL-9 plays a pivotal role in providing immunity against helminth infections by expelling worms *via* enhanced intestinal muscle contraction, mucus production, and increased mast cell activity (52). Th9 cells provide immunity against *Nippostrongylus brasiliensis*, as adoptive transfer of Th9, but not Th2, cells into Rag-deficient hosts provided long-lasting immunity against worms (53, 54). Similarly, animals expressing dominant negative form of TGFβRII in CD4^+^ T cells were found to have reduced levels of IL-9 associated with enhanced parasitic burden in *T. muris* infection (10).

In addition to inducing immunity against extracellular pathogens as well as tissue inflammation in organ-specific autoimmunity, Th9 cells were also found to be associated in mounting superior antitumor response as compared to Th1 and Th17 cells (24). In fact, *Il9* SNP was found to be linked with enhanced risk of cutaneous malignant melanoma (55). In a tumor microenvironment, Th9 cells were found to produce CCL20, which facilitate the migration of CCR6^+^ leukocytes in the tumor tissue. Moreover, IL-3 and IL-21 produced by Th9 cells, respectively, promote dendritic cells survival and functions as well as CD8^+^ CTLs (56). Adoptive transfer of antigen-specific Th9 cells in B16-F10 melanoma models reduces tumor burden and severity, and this antitumor effect of Th9 cells is found to be dependent on IL-9, as neutralization of IL-9 suppressed the antitumor functions of Th9 cells (24). On an intriguing note, adoptive transfer of IL-1β preconditioned Th9 cells retained their antitumor functions in IL-9R-deficient mice or upon IL-9 neutralization in wild-type mice *in vivo* (24). Although multiple studies have demonstrated the potent antitumor functions of Th9 cells, however, the protective effect of Th9 cells in tumor was found to be restricted to solid tumors such as melanoma and lung-adenocarcinoma (57, 58).

Although Th9 cells were known to be involved in multiple diseases, the *in vivo* differentiation and development of Th9 cells is not well defined. Most of the studies have defined the early events in Th9 cells, suggesting a caveat in understanding of the precise genetic programming involved in stably developed Th9 cells. Recently, it has been shown that *in vitro* differentiated Th9 cells lose their ability to secrete IL-9 with chronic stimulation, suggesting that Th9 cells transiently produce IL-9 during *in vitro* differentiation (59). The loss of IL-9 production in Th9 cells could also be explained in the context of Th plasticity, as Th9-Th1 plasticity was suggested (51). Adoptively transferred MOG-specific Th9 cells were found to be converted into IFN-γ-producing cells at sites of target tissues (51). Similarly, Th9 cells produce copious amounts of IFN-γ in B16F10 melanoma model. These observations clearly suggest that Th9 to Th1 plasticity may be crucial for inducing effector functions in these disease models (51). However, none of the studies molecularly defined the Th9 plasticity in greater details.

Although IL-9 is predominantly produced by Th9 cells, however, the production of IL-9 is not restricted to Th9 only. Other effector and regulatory T cells such as Th2, Th17, Tfh, and Tregs cells also produce IL-9 (3, 4, 60–64). It is possible that, in general, during TCR-dependent stimulation, epigenetic modifications keep *Il9* locus accessible for regulators for a narrow time frame. It can also be suggested that a common transcriptional signature might be shared by all these subsets leading to expression of *Il9* by T cells. The transcriptional profiling of Th9 cells suggests the involvement of multiple transcription factors, however, Th9 cells is still devoid of its master regulator as compared to other Th subsets (20, 23, 62). Nonetheless, the presence of IL-9^+^ T cells in patients and target tissues suggests their importance and development *in vivo*.

## Phosphoinositide 3-Kinase (PI3K) Signaling and Role of Foxo1 Transcription Factor in CD4^+^ T Cells

Phosphoinositide 3-kinase signaling plays crucial role in integrating diverse biological functions ranging from cell survival, metabolism to tolerance and aging. In response to growth factor stimulation, PI3K activation transmit cellular signals and further activate Akt and mTOR signaling pathway, which together contributes to different biological processes such as tumor survival including angiogenesis and recruitment of inflammatory cells (65–67). Though there are four different classes of PI3K, but class IA and class IB are elucidated in detail in activation and functions of T cells (67). The major function of these kinases is to activate PLCγ to generate PI3K effector molecules of cell signaling such as diacyl glycerol and inositol-1, 4, 5-triphosphate (IP3), which induces calcium mobilization, and thus leading to PKC activation and NF-κB nuclear translocation. PI3K also phosphorylate PtdIns (4,5) P2 to generate PtdIns (3,4,5) P3, which in turn activates downstream kinases specifically with PH domains such as Akt. Upon T cell activation, Akt, a serine threonine kinase, gets phosphorylated at Thr308 and Ser473 by PDK1 and mTORC2, respectively, to attain its complete activation (65–67). Although PI3K axis has been shown to be essential for clonal expansion and differentiation of Th1 and Th17 cells, however, PI3K negatively regulates regulatory T cells development and function, as TGF-β1-induced Foxp3 expression is impaired upon constitutive AKT activation (68–70). Transcription factor Foxo1 is one of the major downstream targets of Akt activation and regulates cell cycle, cell survival, and energy generation. Foxo transcription factors were shown to respond to variety of physiological stimuli and physiological conditions including oxidative stress, mitogenic factors, and inflammation (71). In lymphocyte compartment, Foxo factors regulate T cell homing and homeostasis, formation of memory T cells, and process of T cell differentiation (72, 73). Transcriptional activity of Foxo factors is regulated by various posttranslational modifications which are collectively known as Foxo code (71). In a simplified context, upon growth factor stimulation, Foxo gets localized to cytoplasm subsequent to phosphorylation at three conserved residues (T24, S256, and S319) by Akt or serum glucocorticoid kinase-1 (SGK1) kinase and subsequently leads to nuclear export and degradation (71).

Due to their dominant role in controlling T cell survival, migration, and metabolism, Foxo1 is studied in most of the effector and Tregs as well as in CD8^+^ T cells (Table 1). Within CD8^+^ T cells, Foxo1 suppresses IL-12-dependent T-bet expression while promotes memory CD8^+^ T cells phenotype by inducing expression of Eomes. Although Foxo1 have binding sites in T-bet promoter, it does not bind to T-bet promoter and exerts its functions in DNA-binding independent manner (74, 75). Foxo1-deficient CD8^+^ T cells expand normally and form effectors, but failed to make pool of memory cells (74–76). Inhibition of glycolysis in CD8^+^ T cells facilitates nuclear localization of Foxo1 and enhanced expression of Foxo1 target genes such as *Klf2, Cd62l* (L-selectin), *Ccr7* (chemokine receptor), and *S1p1r* (sphingosine-1-phosphate receptor 1) (77). It was shown that PI3K/Akt/Foxo1 axis is considered to be a major pathway involved in Tregs to Th1 reprogramming (68). Metabolic regulator PPARγ is also known to stabilize Foxo1 functions, as PPAR-γ-deficient CD4^+^ T cells were found to produce enhanced levels of pro-inflammatory cytokines such as IFN-γ and IL-17 due to inhibition of Foxo1 functions (78). In addition to Th1 and Th17 cells, Foxo1 has been shown to negatively regulate the development of T follicular (Tfh) cells, which are marked by the expression of *Bcl6* and CXCR5 (79).

**Table 1:** Role of Foxo1 in effector and regulatory T cells.

Cell types	Functional role of Foxo1	Reference
Helper T (Th)1	Foxo1 inhibits T-bet-dependent differentiation program of Th1 cells. Foxo1^−/−^CD4^+^ T cells adopt T-bet^+^IFN-y^+^ phenotype upon TGF-β1 induction. Phosphoinositide 3-kinase/Akt/Foxo1/3 pathway is considered to be a dominant signaling axis involved in human Tregs to Th1 reprogramming	(68, 74–76)
Th2	Although role of Foxo1 is not directly investigated in Th2 cells, we have shown that Foxo1 is required for interleukin (IL)-9 induction in Th2 cells without affecting IL-4 production	(62)
Th9	Three independent concordant reports have shown that Foxo1 is essential for IL-9 production in Th9 cells	(16, 62, 94)
Th17	Foxo1 reciprocally regulates IL-9 and IL-17 production in Th17 cells, as Foxo1 suppresses IL-17 but enhances IL-9 in Th17 cells.	(62, 80–82)
Induced Tregs (iTregs)	Foxo1 is required for IL-9 induction in TGF-β1-induced Tregs *in vitro*. Foxo1^−/−^ naïve CD4^+^ T cells display defective differentiation toward iTregs	(62, 84, 85, 96)
Natural Tregs	Decreased frequency of Foxp3^+^ natural Tregs were seen in the thymus of mice harboring conditional deficiency of Foxo1 in Foxp3^+^ and/or CD4^+^ T cells. These Foxo1 conditional deficient mice develop gross immune-pathology, as Foxo1 is required for both development and function of nTregs	(83–85)
T follicular cells	Genetic deletion of Foxo1 favors the development of Tfh cells. CXCR5^+^PD-1^+^ Tfh cells accumulate in large numbers in mice with T cell specific deletion of Foxo1. Foxo1 negatively regulates Bcl-6, one of the major transcription factor required for Tfh differentiation	(78, 79)
CD8^+^ T cells	Foxo1 negatively regulates the T-bet mediated type I effector differentiation while promotes the development of memory CD8^+^ T cells	(74–76)

Foxo1 is a potent suppressor of both human and mice Th17 differentiation (69, 80–82). γ_c_ cytokines such as IL-2, IL-7, or IL-15 are known to drive IL-17 and IL-22 expression in CCR6^+^ human memory T cells as compared to CCR6^−^ T_m_ cells. Furthermore, γ_c_ cytokines activate PI3K axis and represses Foxo1 thereby promoting human Th17 differentiation (69). Consistently, ectopic expression of Foxo1 suppresses γ_c_-mediated IL-17/IL-22 expression in CCR6^+^ T_m_ cells (69). In addition to PI3K, Foxo1 is also regulated by SGK1 *via* regulating IL-23–IL-23R signaling, which is essential for stabilizing and acquiring the pathogenic functions of Th17 cells (80). IL-23R–SGK1 axis has been shown to suppress Foxo1 transcriptional activity by inducing its phosphorylation. In addition, Foxo1 is not only a potent inhibitor of Rorγt-mediated transactivation of *Il23r* expression but also binds directly to RORγt *via* DNA-binding domain (DBD) thereby suppressing Rorγt dependent transcriptional program of Th17 cells (81). In fact, Foxo1 T cell conditional deficient mice have shown increased numbers of Th17 cells in thymus and periphery as compared to wild-type mice (81). Furthermore, in a mixed bone marrow chimera experiment, Foxo1 deficiency is sufficient to drive Th17 differentiation *in vivo* upon antigen challenge as compared to wild-type cells (81). It has been demonstrated that dicer-regulated microRNA-183-96-182 (mir-183C) regulates pathogenic Th17 differentiation *via* suppressing Foxo1 (82). Hence, proposing that factors which are positively associated with Foxo1 are the negative regulators of Th17 differentiation.

As compared to its role in effector CD4^+^ T cells, Foxo1 functions are well established in induction and functions of Tregs (83–86). Foxo1-deficient mice were shown to have expanded population of CD4^+^CD44^hi^ T cells as well as hyper B cell activation leads to hyper gammaglobulinemia and expansion and increased number of follicular T cells. Although the frequency of thymic Foxp3^+^ Tregs was found to be decreased in mice that harbor Foxo1 conditional deficiency in Foxp3^+^ Tregs, the frequency of Foxp3^+^ Tregs in the periphery remain normal in these mice. Nonetheless, Foxo1 deficiency in Tregs leads to lose their suppressive ability (83, 84). In addition to Foxo1, Foxo3 is also expressed in immune cells and Foxo3^−/−^ mice do not develop spontaneous autoimmunity or purified T cells have no defect in proliferation or survival. Combined deletion of both Foxo1 and Foxo3 in T cells leads to fatal systemic inflammatory disease due to defective Foxp3 Tregs development (85), suggesting a specific involvement of Foxo1 in regulating Foxp3 Tregs functions and development. Furthermore, Foxo1 facilitate the binding of other transcription factors at Foxp3 locus thereby regulating complete Foxo1-mediated transcriptional gene program (83, 85, 87). Overexpression of Akt, a negative regulator of Foxo1, has been shown to inhibit suppressive function of Tregs (68, 84). Functionally, Tregs are also classified as resting Tregs, found in spleen and lymph nodes, and activated Tregs found in lymphoid organs and non-lymphoid tissues. Recently it has been demonstrated that Foxo1 repression is associated with enhanced migration of activated Tregs to tumor sites while Foxo1 gain of function leads to quick depletion of activated Tregs, resulting in effective tumor immunity (86). Taken together, Foxo1 have a discrete role in effector and regulatory T cells development and functions.

## Foxo1 Regulates IL-9 Production and Development of Th9 Cells

Th9 cells are now unraveled as a separate subset of effector CD4^+^ T cells and one of the dominant producers of IL-9 (8, 9). Emerging literature has suggested the involvement of various transcription factors in the development of Th9 cells, as Th9 cells emerges as an effector Th population involved in the pathogenesis of many diseases like allergy, asthma, IBD, and antitumor immunity (20, 23, 62). Although previous reports have demonstrated the involvement of IRF-4, PU.1, BATF, and IRF-1 in induction and functions of Th9 cells, none of these factors determine the lineage-specificity Th9 cells. In fact, most of these transcription factors (IRF-4, PU.1, BATF, and IRF-1) are also shared by other Th cells—for example, IRF-4 and PU.1 expressed in Th2 cells, IRF-4 and BATF expressed in Th17 cells, and IRF-1 expressed in Th1 and Tr1 cells (88–93). We and others have recently characterized that Foxo1 is essential for Th9 and other IL-9-producing T cells (16, 62, 94). Though Foxo1 is also co-expressed or shared by regulatory T cells, nonetheless Foxo1 generally suppresses other effector lineages such as T-bet/Th1 cells and Rorγt/Th17 cells. Unlike other transcription factors of Th9 cells (IRF-4, BATF) which also promote the production of IL-4, IL-17 by T cells, Foxo1 negatively regulates the production of IFN-γ and IL-17 with no observable effect on IL-4 induction. This suggests that Foxo1 may not be a unique transcription factor of Th9 cells but does impart specificity in promoting IL-9 induction in Th9 cells as well as in other T cells (75, 77, 78, 80, 81, 93).

As discussed above that PI3K/Akt axis generally promotes the effector function of CD4^+^ T cells while suppresses regulatory T cell development, however, its role in IL-9 induction is lately elucidated. Inhibition of upstream PI3K/AKT by pan-PI3K inhibitor (LY294002) pathway enhanced the induction of IL-9 in Th9 cells. Since Foxo1 tends to be one of the major downstream cellular targets of PI3K/Akt axis, therefore inhibition of PI3K axis results in reduced levels of phospo-Foxo1, mark of inactive cytosolic form of Foxo1 (62). Furthermore, inhibition of Foxo1 reversed the effects of PI3K/AKT inhibition on Th9 cells, suggesting the previously uncharacterized involvement of PI (3)K/AKT-Foxo1 axis in inducing the development of Th9 cells (62). In fact, time kinetics of Foxo1 expression in Th9 cells suggests that it is induced early starting from 24 h of differentiation and is maintained till 72 h (16). Akin to Foxo1, mTOR is also targeted by PI3K/Akt. Suppression of mTOR by rapamycin or stimulation of mTOR by mTOR activator MHY1485 substantially inhibited or promoted Th9 differentiation, respectively (16). Similar to overexpression of constitutive form of Akt, Th9 differentiation was found to be compromised in Foxo1-deficient CD4^+^ T cells or upon direct inhibition of Foxo1 by chemical or genetic approaches (16, 62, 94). In addition of PI3K/Akt, TGF-β1-induced Smad3 pathway is implicated to be involved in Foxo1 induction in Th9 cells (94).

**Figure 1:**
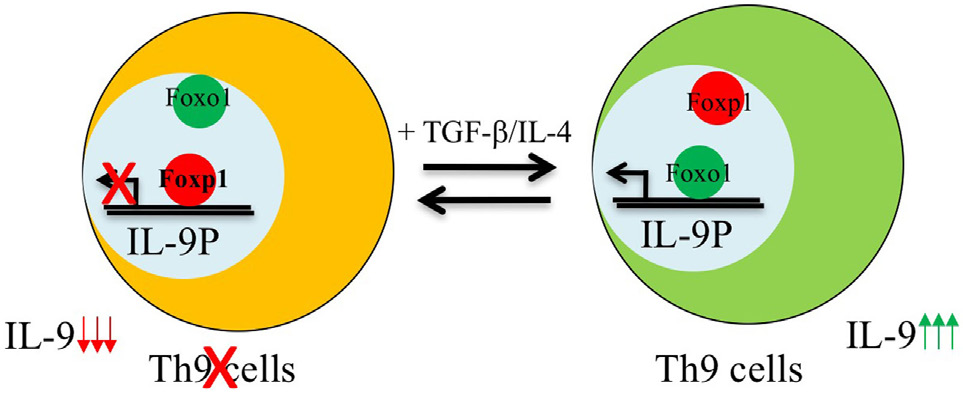
Reciprocal regulation of Foxo1 and Foxp1 in the development of Th9 cells. Foxp1 is enriched and bound to interleukin (IL)-9 promoter in naive CD4^+^ T cells. Activation of naive T cells in the presence of Th9 polarizing cytokines induces Foxo1-co-activator p300 which recruits Foxo1 from cytoplasm to nucleus leading to displacement of Foxp1 from Foxo1 binding sites as both these factors bind to the same region in IL-9 locus, and therefore induces the development of Th9 cells.

Similar to IL-1β-induced IRF-1 expression in Th9 cells, Foxo1 is seen to be induced upon IL-7 stimulation during Th9 differentiation (16, 24). Priming of naïve CD4^+^ T cells with IL-7 not only enhances *Il9* and *Il21* expression but is also essentially required for repressing the Th9 repressor-Foxp1 (16). During Th9 differentiation, both Foxo1 and Foxp1 are competitively regulated as both these Fox members have similar conserved binding site on IL-9 promoter. IL-7 priming of Th9 cells induces p300 which is both a co-activator and stabilizer of Foxo1 protein (16). This not only enables Foxo1 to outcompete Foxp1 for binding to IL-9 promoter but also leads to decreased amounts of phosphorylated Foxo1 protein in CD4^+^ T cells thereby increasing the relative amounts of total Foxo1 protein (Figure 1). Due to redundancy of Foxo proteins, in addition of Foxo1, Foxo4 was also found to augment Th9 differentiation and provides a link to the observation as why noticeable Th9 differentiation is still observed in Foxo1 conditional deficient CD4^+^ T cells (16).

While being in nucleus, Foxo1 not only targets the cytokine gene locus in order to directly regulate the lineage specific cytokines but also auto-amplifies itself, as Foxo1 can bind to its own promoter (95). Since Foxo transcription factors have both DBD and protein interaction domain, therefore they mediate their function in both DNA dependent and independent manner (71). Foxo1 physically interact with IL-9 and IRF-4 promoters in Th9 other IL-9-producers such as Th17 and iTregs (62). Moreover, DNA-binding activity of Foxo1 is required for IL-9 induction in Th9 cells, as ectopic expression of Foxo1 that lacks DBD fails to enhance IL-9 in Th9 cells. Interestingly, both overexpression and inhibition of Foxo1 in Th9 cells, respectively, enhances or suppresses other Th9-associated genes such as IRF-4, PU.1, BATF, and IRF1, indicating a potential involvement of Foxo1 partner complexes in regulating IL-9 induction. In fact, in an *in vitro* protein interaction assay Foxo1 was found to interact with IRF-4, suggesting that Foxo1–IRF4 protein complex might be essential for the induction of Th9 cells (62). Since modulation of Foxo1 also resulted in changes in Th9 genetic program, hence it could be hypothesized that Foxo1 is induced early during Th9 differentiation and may act upstream of other factors, which are crucial for development of Th9 cells. Corroborating this, Foxo1 directly induces and acts upstream of IRF4 in Th9 cells and thereby potentiating the development of Th9 cells (62, 94). It could be suggested that similar to IRF-8, which participate in formation of transcriptional complexes, Foxo1 might also work collectively by recruiting other transcription factors and form large transcriptional complex, which drive optimal *Il9* transcription in Th9 cells (26). In nutshell, above mentioned observations suggested a crucial role of Foxo1 in inducing IL-9 during *in vitro* differentiation of Th9 cells.

As discussed above, IL-9 plays an indispensable role in inducing and promoting atopic diseases such as dermatitis and allergic asthma. Both IL-9 and IL-9R polymorphism are found to be genetically associated with asthma. Consistently, circulating T cells from allergic patients have enhanced capacity to produce IL-9 in response to pollen or house dust mite extract (5, 37). As reported by fate reporter system, Th9 cells are one of the major IL-9 producers in OVA sensitized mice models. In fact, administration of anti-IL-9 neutralizing antibody after allergen sensitization reduced allergic inflammation in murine model of asthma associated with attenuation in inflammatory cell infiltration, indicating the crucial role of IL-9 in development of asthma. Since our *in vitro* data suggests a strong association of IL-9 and Foxo1, hence we speculated if Foxo1 also regulates IL-9 *in vivo* during the pathogenesis of asthma. Corroborating *in vitro* data, *in vivo* blockade of Foxo1 resulted in reduced signs of asthma as measured by AHR and associated with reduced frequency of IL-9^+^CD4^+^T cells in the lungs (62, 94). In a B16-OVA tumor model, IL-7-treated OT-II Th9 cells were found to mount potent antitumor activity, which is suppressed in the absence of Foxo1, suggesting that Foxo1-mediated IL-9 induction is essential in mounting the potent antitumor immunity by Th9 cells (16).

Since Foxo1 inhibition in asthma model reduced IL-9-dependent inflammation and antitumor potential of Th9 cells, hence targeting Foxo1 could provide a potential therapeutic advantage in these diseases. Nonetheless, due to lack of firm understanding of the time window at which IL-9 appears *in vivo*, it is difficult to extrapolate when and how Foxo1 controls IL-9 appearance *in vivo*.

## Foxo1 Tunes IL-9 Induction by Th17 Cells and Regulatory T Cells

Interleukin-9 is a pleotropic cytokine and its expression is not confined to one particular T cell subset. IL-9 is shown to be produced by Th2, Th9, Th17 cells, and Tregs. Interestingly, IL-9 is produced by Th17 cells induced by TGF-β1/IL-6. Further detailed analysis of pathogenic and non-pathogenic Th17 cells revealed that IL-9 production is restricted to non-pathogenic Th17 cells (polarized with TGF-β1/IL-6), as pathogenic Th17 cells induced by IL-23 exposure lose IL-9 production (63, 80). In addition to IL-23, pathogenic Th17 cells are also induced by TGF-β3 and IL-1β combined separately with IL-6. Interestingly, TGF-β3 and IL-6 polarized Th17 cells express less *Il9* as compared to TGF-β1 and IL-6 (62). Cogently, pathogenic Th17 cells maintain high levels of phospho-Foxo1 during their differentiation suggesting the sequestration and degradation of Foxo1 in cytoplasm. Though cytosolic Foxo1 is suggested to be marked for degradation, however, cytosolic Foxo1 regulates autophagy and is not truly non-functional in cytoplasm (96). In addition of PI3K/Akt, Foxo1 is also negatively regulated by salt sensing kinase SGK1. Strikingly, IL-9 is one of the highly expressed genes in SGK-deficient Th17 cells as compared to wild-type Th17 cells. Interestingly, SGK1 also promotes the generation of pathogenic Th17 cells thus endorsing the fact that non-pathogenic Th17 cells not only express *Il9* but also *Foxo1* while acquisition of pathogenicity by Th17 cells leads to concomitant disappearance of both *Il9* and *Foxo1* (62).

**Figure 2:**
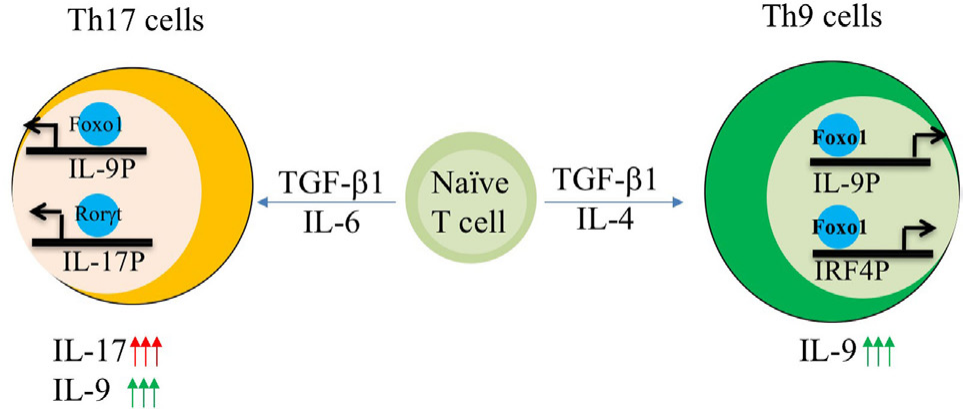
AKT–Foxo1 axis regulates interleukin (IL)-17, IL-9 induction in Th9 and Th17 cells. Fine tuning of Akt–Foxo1 axis determines the production of IL-9 in both Th9 and Th17 cells. Upon phosphoinositide 3-kinase (PI3K)/Akt inhibition, Foxo1 is induced in Th9 cells, which in turn binds to both IL-9 and IRF-4 promoter thereby contributing to optimal expression of IL-9 in Th9 cells. Unrestrained activity of Akt regulates IL-17 production by Th17 cells. Inhibition of PI3K/Akt axis suppress IL-17 while enhances IL-9 in Th17 cells leading to a switch from high IL-17 producers to high IL-9 producing Th17 cells.

Since Foxo1 is generally controlled by PI3K/Akt axis, hence we employed a reverse approach where inhibition of PI3K by pan-inhibitor or overexpression of dominant form of Akt not only suppressed IL-17 and Th17 genetic program but also enhanced the induction of IL-9 in Th17 cells. Similarly, inhibition of Foxo1 in Th17 cells also restrains the production of IL-9 (Figure 2). While suppression of IL-17 by Foxo1 is not surprising, as Foxo1 is previously known to directly inhibit RORγt (81). However, what is truly compelling is the ability of Foxo1 to reciprocally regulate the balance of IL-9 and IL-17 in Th17 cells (62). Though it is not known what drives the expression of IL-9 in Th17 cells, physiological need to secrete IL-9 or if IL-9 production by Th17 cells is a transient phenomenon. Nonetheless, current study suggests that Foxo1 can discern *Il9* expression over *Il17* in Th17 cells (62).

Strikingly, not only effector T cells but regulatory T cells also produce IL-9. While molecular mechanisms driving the induction of IL-9 in Tregs is not known, however, gene expression analysis have shown the transcriptional similarities between Th9 and Tregs due to the presence of shared differentiation factor TGF-β1 in culture conditions (21). Functionally, IL-9 production by regulatory T cells is seen to mediate protective immunity against nephritis in a model of nephrotoxic serum nephritis (NTS) and skin allograft tolerance *via* Treg–mast cell cooperation in the target organ during inflammation (64). Moreover, IL-9R-deficient mice immunized with MOG develops severe EAE and Tregs isolated from IL-9R-deficient animals were shown to have poor suppressive function, suggesting that IL-9 regulates Tregs suppressive functions in autoimmunity (60). Since we have found an association of Foxo1 with IL-9 in effector T cells, therefore we speculated whether Foxo1 is also required for IL-9 induction in Tregs. We have reported that TGF-β1-induced iTregs not only express *Il9* but also other Th9-associated factors such as *Batf, Irf4* and *Spi1* and *Klf2*, a Foxo1 target gene, and inhibition of Foxo1 in iTregs leads to IL-9 suppression. Interestingly, Foxo1 physically binds to IL-9P in iTregs thereby regulating *Il9* transcription directly. The ChIP binding data also suggests that Foxo1 has accessibility to IL-9P during TGF-β1 permissive milieu, however, if Foxo1 is involved in directly modulating chromatin dynamics at IL-9P is not known. Though this preliminary data only suggests that Foxo1 plays a functional role in IL-9 induction by iTregs. Furthermore, in order to prove an essential requirement of Foxo1 for IL-9 induction in Tregs, experiments employing Tregs isolated from Foxp3^cre^Foxo^fl/fl^ conditional deficient mice or Foxp3^cre^Foxo1^AAA^ (Akt-mediated phosphorylation site is mutated) should be implemented. Since Tregs express other Th9-associated factor such as IRF-4, hence we do not exclude the possibility of involvement of other transcription factors in regulating IL-9 induction by Tregs. The production of IL-9 by regulatory T cells and IL-10 producing Th17 cells suggests that IL-9 might play a significant role in regulating inflammation *via* immune-suppression. In a striking contrast, murine Th9 cells also produce IL-10 but lacks immune-suppressive capability thereby enforcing the idea of a subset specific role of IL-9 in inducing or ameliorating tissue inflammation.

## Conclusion and Perspective

Over the last one decade since the discovery of Th9 cells in 2008, an extensive array of signaling axis, transcription factors, and physiological functions that are involved in development and amplification of IL-9^+^ T cells have been unraveled. Very recently, growth factor-dependent PI3K/Akt axis *via* Foxo1 is seen to control the development of IL-9 production by effector T cells. Strengthening the role of Foxo1, three independent groups have summarized the essential requirement of Foxo1 in regulating IL-9 in Th9 cells. It is interesting to note that in general Foxo1 negatively regulates other effector subsets but is seen to be a positive regulator of IL-9 expression. Physiologically, Foxo1-dependent functions are critically required for inducing allergy asthma and antitumor immunity in mouse models. Due to our limited understanding for pathways involved in development of human Th9 cells, ambiguity and overlap of current mechanisms required for Th9 transcriptional regulation with other T helper subsets, the therapeutic exploitation of Th9 cells for targeted therapy against various diseases is still not achieved. Furthermore, a lot more efforts need to be invested in understanding the requirements for maintaining stability of Th9 cells *in vivo*, which will provide a better template for manipulating Th9 cells therapeutically.

## Author Contributions

AA and SM have written and edited the review.

## Conflict of Interest Statement

The authors declare that the research was conducted in the absence of any commercial or financial relationships that could be construed as a potential conflict of interest. The reviewer PP and handling Editor declared their shared affiliation.
